# Supplementary material for: Region-specific genomic variation and functional divergence of Helicobacter pylori clinical isolates from the gastric antrum and corpus
Source: mSystems. 2025 Sep 22;10(10):e01029-25. doi: 10.1128/msystems.01029-25 (PMC12542646; doi:10.1128/msystems.01029-25)
Supplement: Supplemental material — Supplemental figures and table. [file msystems.01029-25-s0001.pdf]

Supplemental Material

Table S1. Genetic comparison between antrum and corpus strains.

	Gene [a]	Gene no.[b]	% identity of amino acid	
			v225 vs v226	v290 vs v291
Adhesion	Hop family (major outer membrane protein family)			
	hopZ	HP0009	100	98
	hopD	HP0025	100	99
	hopM	HP0027	100	99
	hopA	HP0229	100	96
	hopF	HP0252	99	100
	hopG	HP0253/0254	100	99
	hopJ	HP0477	none	none
	hopH	HP0638	100	100
	hopE	HP0706	100	99
	hopC	HP0912	100	97
	hopB	HP0913	100	93
	hopK	HP0923	none	none
	hopI	HP1156	99	97
	hopL	HP1157	99	97
	hopQ	HP1177	96	90
	hopN	HP1342	none	none
	hopU	HP0317	none	none
	sabA (hopP)	HP0725	100	none (only 291)
	sabB (hopO)	HP0722	none	none
	babA (hopS)	HP1243	99	63
	babB (hopT)	HP0896	100	59
Motility	Flagellum A (flaA)	HP0601	100	99
	Flagellum B (flaB)	HP0115	100	99
Acid resistance	ureH	HP0067	100	100
	ureG	HP0068	100	99
	ureF	HP0069	100	100
	ureE	HP0070	99	100
	ureI	HP0071	100	99
	ureB	HP0072	100	100
	ureA	HP0073	100	99
	lipoprotein signal peptidase (lspA)(ureC)	HP0074	100	98
	Phosphoglucosamine mutas (glmM)(ureD)	HP0075	100	100
Toxin	cagPAI			
	cag1 (cagζ)	HP0520	100	100
	cag2 (cagε)	HP0521 pseudo	none	none
	cag3 (cagδ)	HP0522	100	100
	cag4 (cagγ)	HP0523	100	100
	cag5 (cagβ)	HP0524	100	100
	cagα	HP0525	100	100
	cagZ	HP0526	100	99
	cagY	HP0527	100	91
	cagX	HP0528	100	100
	cagW	HP0529	100	100
	cagV	HP0530	100	100
	cagU	HP0531	100	100
	cagT	HP0532	100	100
	cagS	HP0534	100	100
	cagQ	HP0535	100	100
	cagP	HP0536	100	100
	cagM	HP0537	100	100
	cagN	HP0538	100	100
	cagL	HP0539	100	100
	cagI	HP0540	100	100
	cagH	HP0541	100	100
	cagG	HP0542	100	100
	cagF	HP0543	100	100
	cagE	HP0544	100	100
	cagD	HP0545	100	96
	cagC	HP0546	100	91
	cagA	HP0547	99	98
	vacA	HP0887	93	89
Others	capJ	HP0421	100	98
	CGAT	HP0499	100	96
	acyl_carrier_protein_(ACP)-dehydratase_CoA	HP0420	100	100
	N-acetyltransferase	HP0935	100	100

[a] Genes for virulence factor of *Helicobacter pylori*.
[b] Based on the *H. pylori* reference strain 26695 as the gene number base.

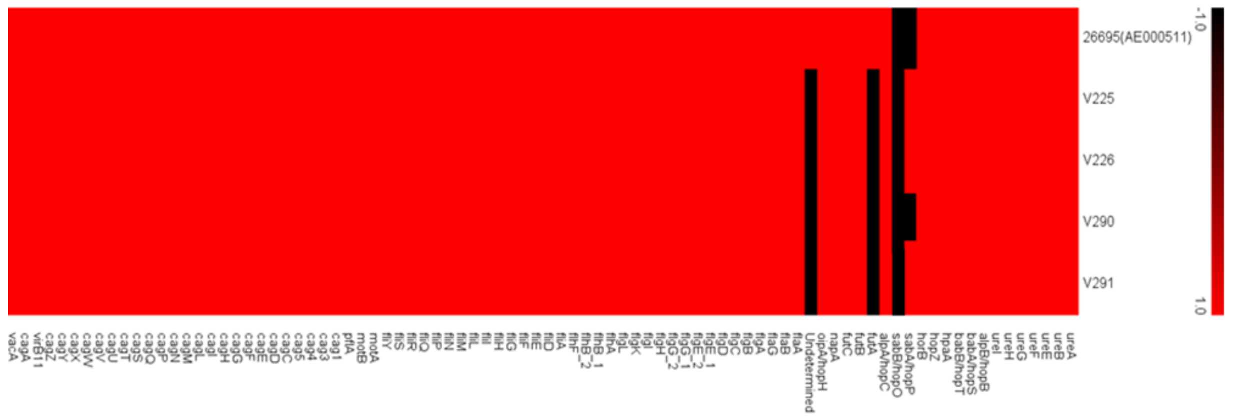

Figure S1. Analysis of virulence genes in *H. pylori* clinical isolates. The *H. pylori* reference strain 26695 (hpNEAfrica1) and clinical isolates v225/v226 and v290/v291 were analyzed for virulence genes related to acid resistance, bacterial adhesion, immune evasion, motility, type IV secretion system, and key toxins. Black blocks indicate deleted genes.

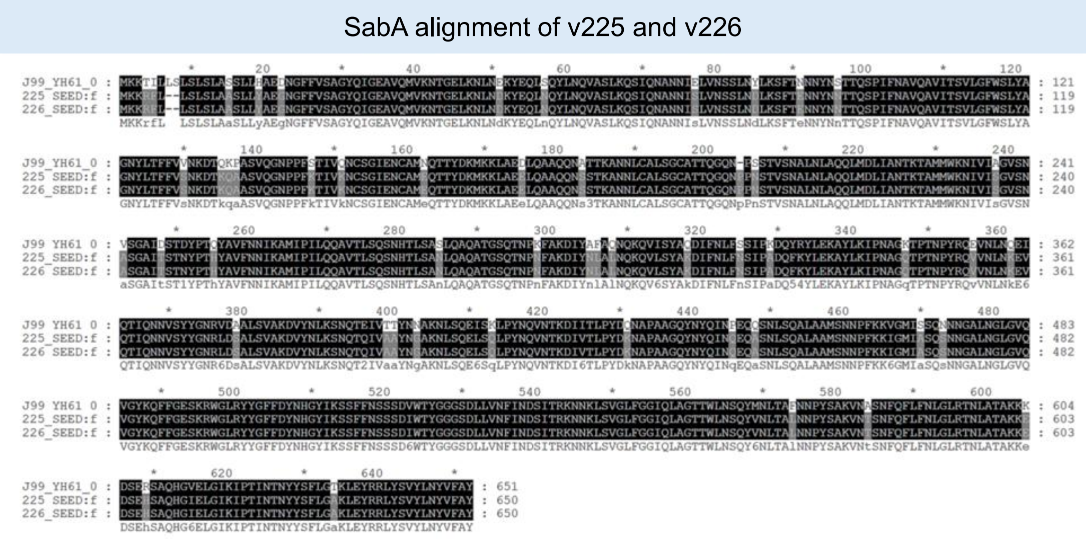

Note：The gene HP_0725 of 26695 contains an authentic frame shift and is not the result of a sequencing artifact

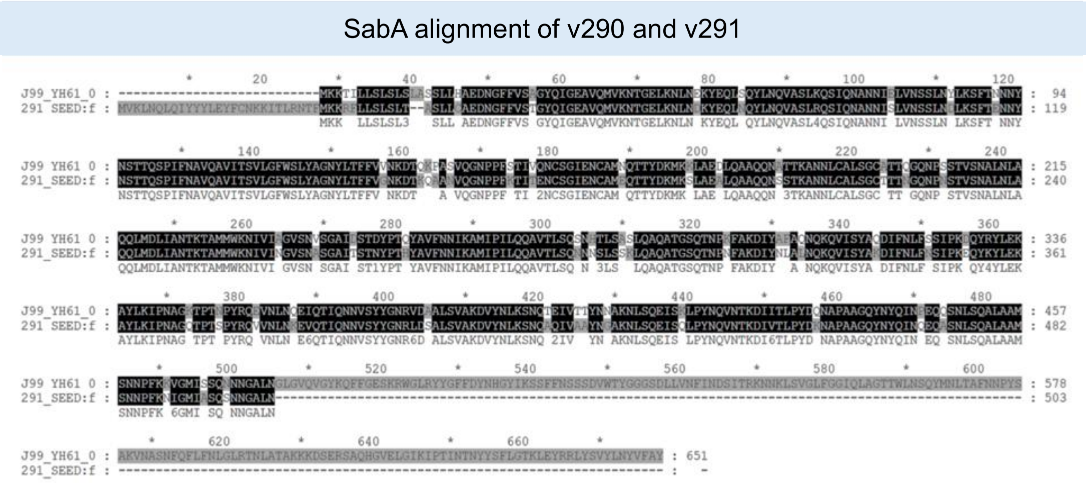

Figure S2. Multiple sequence alignment of SabA from clinical isolates v225/v226 and v290/v291. The gene orientation is based on *H. pylori* reference strain J99. Homologous regions are shaded with black shadows (identical amino acid residues) and gray shadows (conserved amino acid substitutions), and no shading indicates that the residues are neither identical nor similar. Non-coding DNA sequence in *sabA* region of v290.

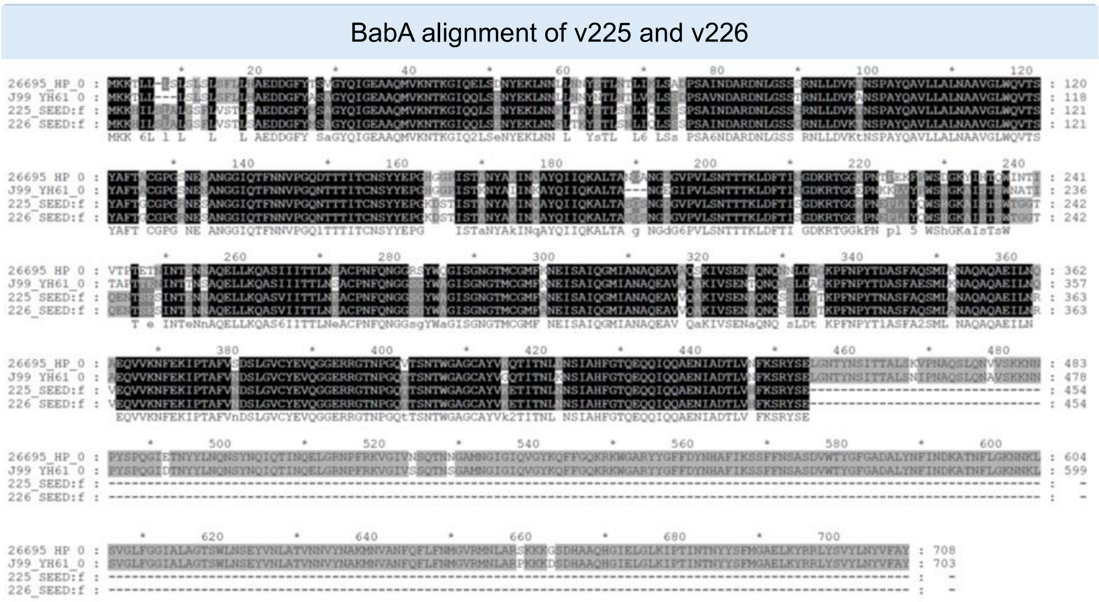

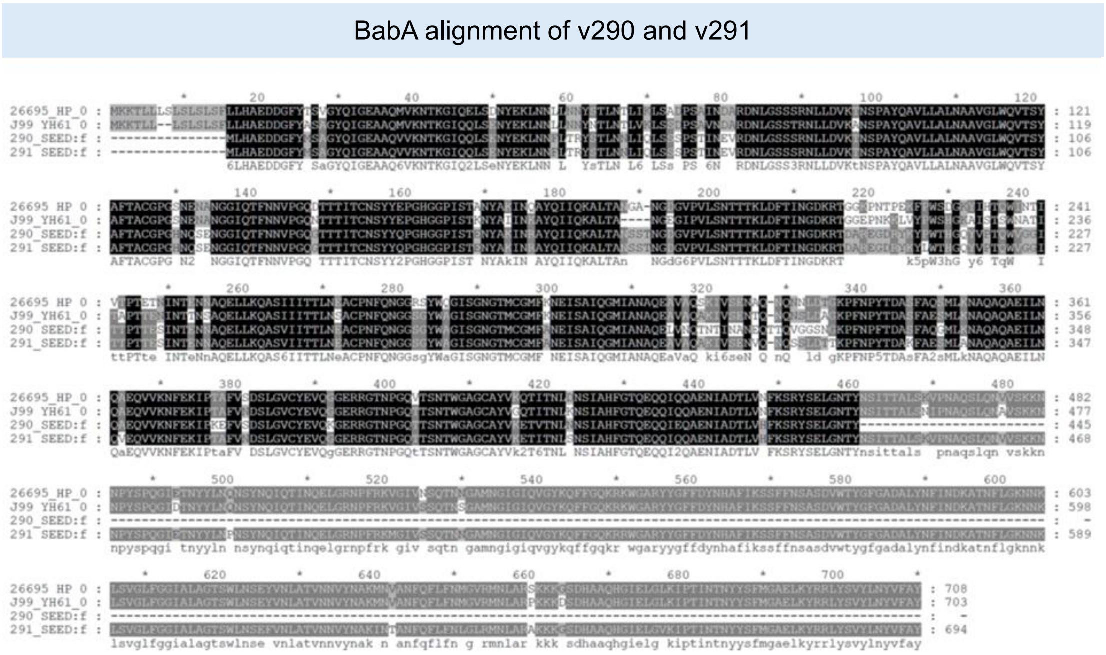

Figure S3. Multiple sequence alignment of BabA from clinical isolates v225/v226 and v290/v291. The gene orientation is based on *H. pylori* reference strains 26695 and J99. Homologous regions are shaded with black shadows (identical amino acid residues) and gray shadows (conserved amino acid substitutions), and no shading indicates that the residues are neither identical nor similar.

BabB alignment of v225 and v226

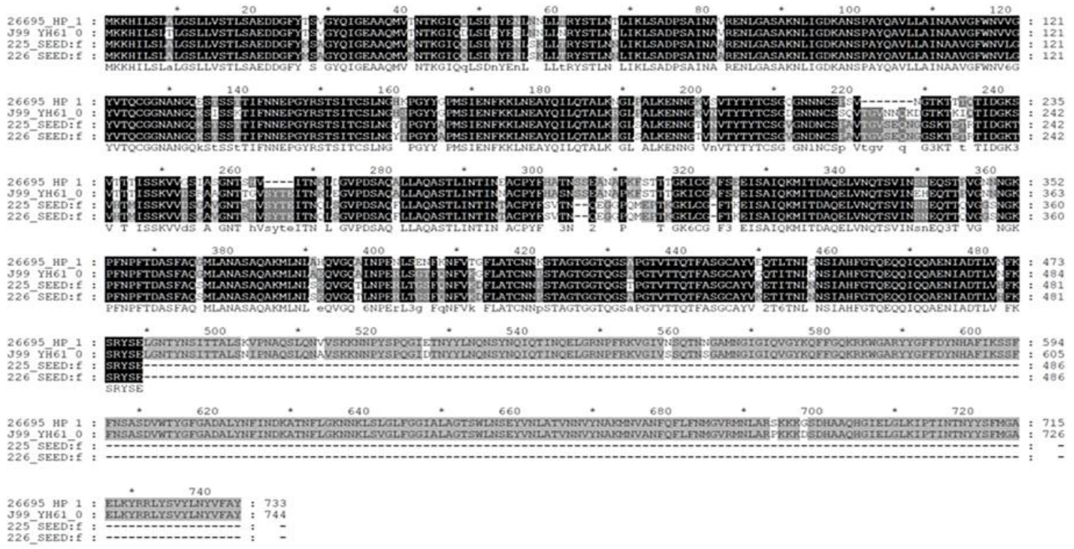

BabB alignment of v290 and v291

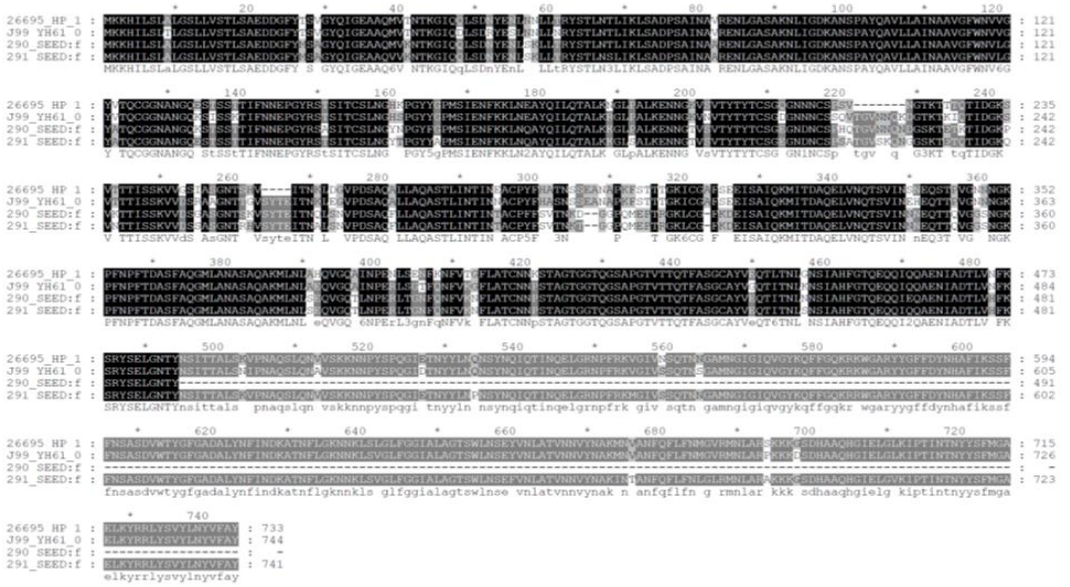

Figure S4. Multiple sequence alignment of BabB from clinical isolates v225/v226 and v290/v291. The gene orientation is based on *H. pylori* reference strains 26695 and J99. Homologous regions are shaded with black shadows (identical amino acid residues) and gray shadows (conserved amino acid substitutions), and no shading indicates that the residues are neither identical nor similar.

CagA alignment of v225 and v226

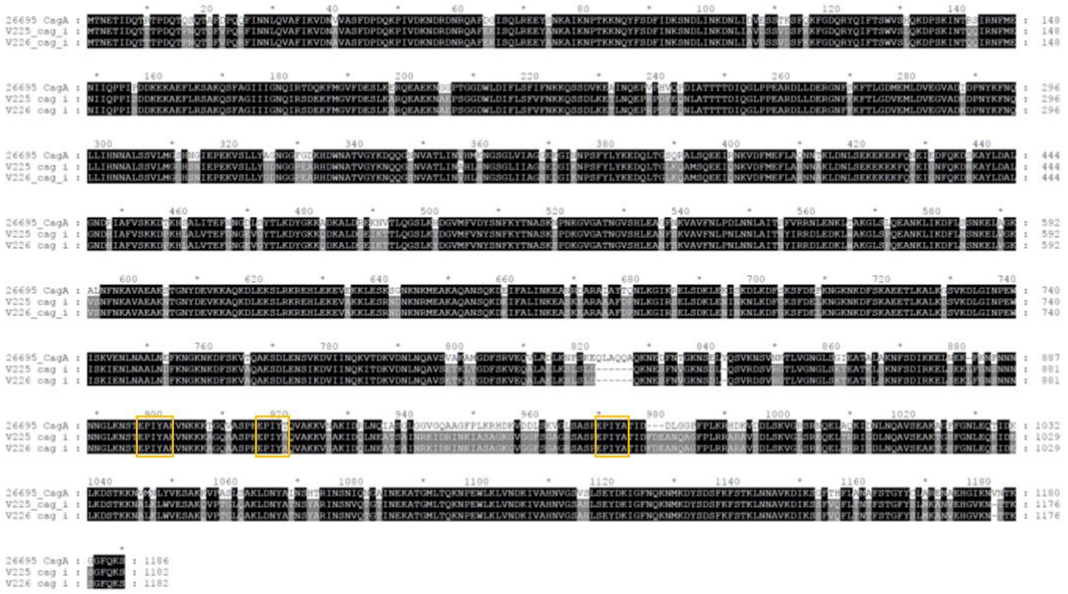

CagA alignment of v290 and v291

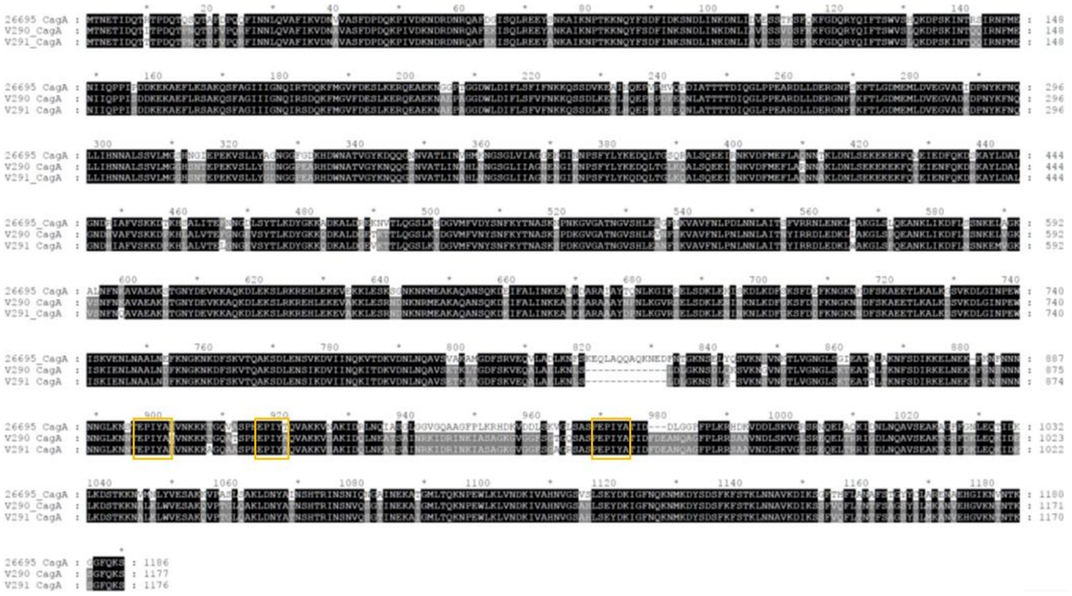

Figure S5. Multiple sequence alignment of CagA from clinical isolates v225/v226 and v290/v291. The gene orientation is based on *H. pylori* reference strain 26695. Homologous regions are shaded with black shadows (identical amino acid residues) and gray shadows (conserved amino acid substitutions), and no shading indicates that the residues are neither identical nor similar.

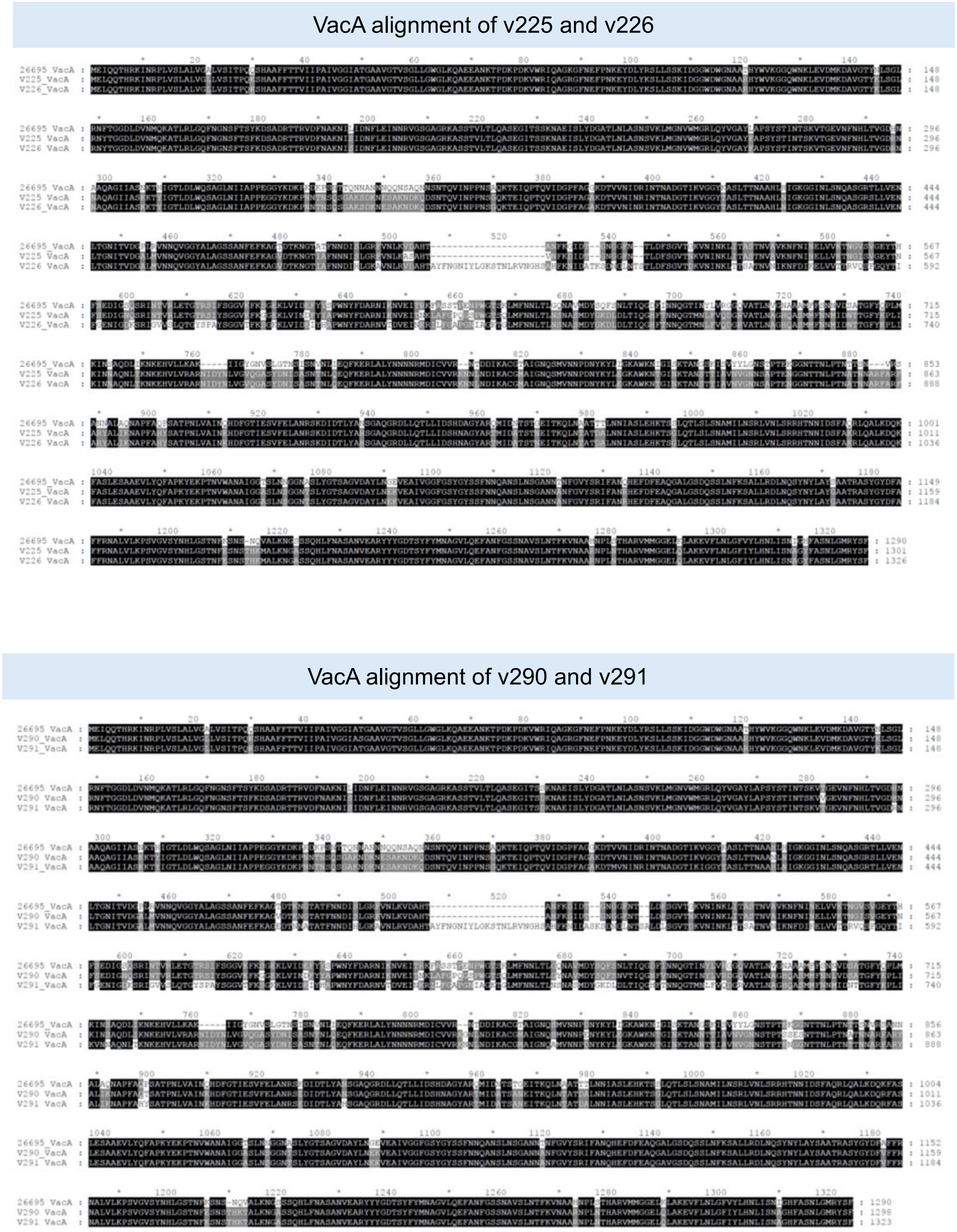

Figure S6. Multiple sequence alignment of VacA from clinical isolates v225/v226 and v290/v291. The gene orientation is based on *H. pylori* reference strain 26695. Homologous regions are shaded with black shadows (identical amino acid residues) and gray shadows (conserved amino acid substitutions), and no shading indicates that the residues are neither identical nor similar.
